# Contrasting physiological adaptation strategies to natural environmental change in two Red Sea coral holobionts

**DOI:** 10.1093/ismeco/ycag008

**Published:** 2026-02-06

**Authors:** Bianca M Thobor, Claudia E L Hill, Gordon F Custer, Neus Garcias-Bonet, Michael D Fox, Yusuf C El-Khaled, Eva Aylagas, Francisco Dini-Andreote, Ulrich Struck, Arjen Tilstra, Raquel Peixoto, Susana Carvalho, Christian Wild, Benjamin Mueller

**Affiliations:** Department of Marine Ecology, University of Bremen, Leobener Strasse 6, Bremen 28359, Bremen, Germany; Department of Marine Ecology, University of Bremen, Leobener Strasse 6, Bremen 28359, Bremen, Germany; Biological and Environmental Sciences and Engineering (BESE) Division, King Abdullah University of Science and Technology (KAUST), Thuwal 23955-6900, Saudi Arabia; Department of Plant Science and Huck Institutes of the Life Sciences, The Pennsylvania State University, University Park, PA 16802, United States; The One Health Microbiome Center, Huck Institutes of the Life Sciences, The Pennsylvania State University, University Park, PA 16802, United States; Department of Natural Sciences, University of Maryland Eastern Shore, MD 21853, United States; Biological and Environmental Sciences and Engineering (BESE) Division, King Abdullah University of Science and Technology (KAUST), Thuwal 23955-6900, Saudi Arabia; Biological and Environmental Sciences and Engineering (BESE) Division, King Abdullah University of Science and Technology (KAUST), Thuwal 23955-6900, Saudi Arabia; Biological and Environmental Sciences and Engineering (BESE) Division, King Abdullah University of Science and Technology (KAUST), Thuwal 23955-6900, Saudi Arabia; Biological and Environmental Sciences and Engineering (BESE) Division, King Abdullah University of Science and Technology (KAUST), Thuwal 23955-6900, Saudi Arabia; Department of Plant Science and Huck Institutes of the Life Sciences, The Pennsylvania State University, University Park, PA 16802, United States; The One Health Microbiome Center, Huck Institutes of the Life Sciences, The Pennsylvania State University, University Park, PA 16802, United States; Museum für Naturkunde, Leibnitz Institute for Evolution and Biodiversity Science, Invalidenstrasse 43, Berlin 10115, Berlin, Germany; Department of Earth Science, Free University Berlin, Malteserstrasse 74-100, Berlin 12249, Berlin, Germany; Department of Marine Ecology, University of Bremen, Leobener Strasse 6, Bremen 28359, Bremen, Germany; Arcadis Nederland B.V, Amsterdamseweg 13, Arnhem, CM 6814, The Netherlands; Biological and Environmental Sciences and Engineering (BESE) Division, King Abdullah University of Science and Technology (KAUST), Thuwal 23955-6900, Saudi Arabia; Biological and Environmental Sciences and Engineering (BESE) Division, King Abdullah University of Science and Technology (KAUST), Thuwal 23955-6900, Saudi Arabia; Department of Marine Ecology, University of Bremen, Leobener Strasse 6, Bremen 28359, Bremen, Germany; Department of Marine Ecology, University of Bremen, Leobener Strasse 6, Bremen 28359, Bremen, Germany; Department of Oceanography and Sea Grant College Program, Center for Microbial Oceanography: Research and Education, University of Hawai’i at Mānoa, 1000 Pope Road, Honolulu, HI 96822, United States

**Keywords:** conformer, *Millepora dichotoma*, nutrient cycling, regulator, trophic plasticity, microbiome flexibility, *Stylophora digitata*

## Abstract

Coral holobionts acquire energy and nutrients from heterotrophic feeding, Symbiodiniaceae symbiosis, and additional metabolic functions (*e.g.* nitrogen (N) fixation) from associated bacterial communities. Since symbioses often require stable environmental conditions, corals in environments with seasonal variability have likely evolved adaptation strategies by either maintaining (i.e. regulating) or shifting (i.e. conforming) key functional traits, but empirical data is needed. We investigated carbon (C) and N elemental and stable isotope ratios alongside bacterial community composition in the hydrocoral *Millepora dichotoma* and the scleractinian coral *Stylophora pistillata* every two months over one year. These data were integrated with environmental parameters to investigate potential adaptation strategies of the coral holobionts over a seasonal cycle. *S. pistillata* showed temporal changes in δ^13^C, δ^15^N and C:N ratios in both host and Symbiodiniaceae tissues (indicating stable host-Symbiodiniaceae C/N cycling), in combination with stable bacterial communities. *M. dichotoma,* did not exhibit temporal changes in elemental and stable isotope ratios, but higher δ^15^N and C:N variability, and 61% higher C:N ratios in Symbiodiniaceae compared to host tissue. Temporal shifts in bacterial communities resulted in significantly enriched predicted metabolic functions for C, N, and sulfur cycling in winter. Stable C/N cycling and bacterial community composition suggest a regulator-like life history strategy of *S. pistillata*, whereas variable C/N cycling and flexible bacterial communities indicate a conformer-like life history strategy for *M. dichotoma*. Both contrasting adaptation strategies enable these organisms to succeed amid current environmental change, yet to what extent this can be maintained under future climate scenarios remains to be investigated.

## Introduction

The symbiosis between heterotrophic corals and phototrophic dinoflagellates of the family Symbiodiniaceae [1] forms the foundation of coral reefs [2], one of the most productive and diverse ecosystems on earth [3, 4]. These endosymbionts share organic carbon in the form of photosynthates with their coral hosts, and in turn, receive shelter, nutrients, and stable environmental conditions [5, 6]. A diverse associated microbial community further adds essential metabolic functions such as N and sulfur (S) cycling, vitamin synthesis, and antimicrobial activities to the functional potential of the coral holobiont (i.e. the animal host and its associated microbiome) [7, 8]. Since the beginning of the Anthropocene, human activities caused a major increase in the severity and pace of environmental changes [9, 10]. Most noticeably, anthropogenic CO_2_ emissions have increased the intensity, extent and frequency of heat waves [11]. However, when environmental parameters exceed the narrow limits coral holobionts have evolved to withstand [12, 13], the delicate balance between holobiont components is disturbed, resulting in dysbiosis [10, 14, 15]. This was prominently showcased in the four global mass coral bleaching events in the past 25 years, threatening the mere existence of coral reef ecosystems worldwide [16, 17].

General ecological theory predicts two response mechanisms of organisms to environmental fluctuations: (i) regulators maintain stable internal conditions through physiological mechanisms (e.g. mammals maintaining stable body temperatures), whereas (ii) conformers display varying internal conditions with environmental change (e.g. body temperatures of reptiles changing with outside temperatures) [18, 19]. This framework can also be applied to the homeostasis of their elemental composition [20], where different coral species can display contrasting strategies in C-N homeostasis [21, 22]. Some coral holobionts appear to regulate N supply to Symbiodiniaceae inside their gastrodermal tissues, ensuring a consistent C:N ratio in Symbiodiniaceae cells [21, 22]. This regulation stabilizes the symbiosis under fluctuating nutrient levels by maintaining N limitation and thus, photosynthate transfer from Symbiodiniaceae to the coral host [15]. Conversely, in corals lacking such regulatory mechanisms, increased environmental N supply can alleviate N limitation for Symbiodiniaceae and destabilize the symbiosis [23]. Both strategies of C/N regulation may be linked to changes in coral-associated bacterial communities, either by contributing to the active regulation of the internal N availability [23], or due to an altered nutrient transfer between Symbiodiniaceae and the coral host [22]. It is currently unclear to which extent microbiome flexibility (i.e. shifts in microbial community composition) relates to the ability of corals to adapt to changing environmental conditions [8] and maintain stable C/N cycling dynamics [23, 24].

Here, we investigated how two dominant reef-building corals, *Millepora dichotoma* and *Stylophora pistillata*, respond to distinct seasonal changes in environmental conditions in the central Red Sea. Hydrocorals of the genus *Millepora* are understudied reef-building organisms [25] that are associated with diverse Symbiodiniaceae communities varying across genotypes [26] and geography, suggesting an adaptable host-Symbiodiniaceae relationship [27]. Similarly, associated bacterial communities are often diverse [28] and can vary with habitat type [29]. We compare this understudied but ecologically relevant [25] reef-building hydrocoral with the well-studied scleractinian coral *S. pistillata* which serves as a model species for coral physiology and ecology [30]. Red Sea *S. pistillata* primarily hosts Symbiodiniaceae of the genus *Symbiodinium*, with fine-scale genetic variation across sites [31]. The less diverse bacterial community of *S. pistillata* is often dominated by the common symbiont *Endozoicomonas* [32]. Despite an intensified variability in seasonal environmental conditions in the Red Sea due to climate change, both species have spread successfully over the past decades [33], suggesting the presence of efficient adaptation strategies that enable them to succeed in an increasingly variable environment.

We aimed to investigate the responses of *M. dichotoma* and *S. pistillata* to natural environmental change in the central Red Sea by either maintaining (i.e. regulating) or shifting (i.e. conforming) their (i) C/N cycling between host and Symbiodiniaceae, and (ii) coral-associated bacterial community compositions. Thereto, we analysed C and N stoichiometry (C: N ratio) and stable isotope ratios (δ^13^C and δ^15^N) of the coral hosts and associated Symbiodiniaceae to assess resource sharing and incorporation of C and N by both holobiont members over one year [22]. We further analysed the composition of bacterial communities associated with both coral species (16S rRNA amplicon sequencing) and predicted their metabolic functions to assess temporal dynamics in potential functions. Results presented here provide insights into the adaptation mechanisms of corals in naturally variable environments, offering a framework to understand how corals may adapt to future environmental conditions.

## Materials and methods

### Sample collection

Sample collections were carried out at Al Fahal Reef (22.30518 N, 38.96468E) in the central Red Sea. This mid-shore reef is located 15 km offshore from the King Abdullah University of Science and Technology (KAUST), Saudi Arabia. Sample collection was performed every two months, in April, June, August, October, and December 2022, as well as February 2023. Colonies of *M. dichotoma* and *S. pistillata* (each *n* = 5) were collected at 2–5 m depth in the first and second week of each sampling month. This collection was part of a larger collaborative sampling effort between KAUST and the University of Bremen including two additional coral species (see Hill et al. [34]). Different colonies were sampled each month, and species were identified visually using Red Sea field guides (see ESM Fig. S10 for representative pictures of the two species). Samples were taken from unshaded colonies at the outer edge of the reef flat, where higher wave energy prevented deposition of sediments. One fragment (~5 cm length) was cut per colony using pliers and placed in seawater-filled sampling bags. These coral fragments were kept on ice during transit and subsequently stored at −20°C at the lab facilities at KAUST until further stable isotope analysis. Additionally, one fragment (~1 cm length) per colony was collected for bacterial community analysis as described above. Fragments were transferred to sterile cone-shaped sample tubes, flash frozen, and stored in liquid nitrogen on the boat, until subsequent storage at −80°C in the lab before processing for 16S amplicon sequencing. Water samples for nutrient analyses (*n* = 2) were taken with hand-held Niskin bottles (5 L volume each) on SCUBA at ~8 m depth at the study site. Aliquots for inorganic nutrient analyses were immediately processed on the boat. Details on environmental chlorophyll and organic and inorganic nutrient sample processing and subsequent analyses are presented in the Supplementary (Text S1).

### Extraction of environmental conditions from satellite data

**Figure 1:**
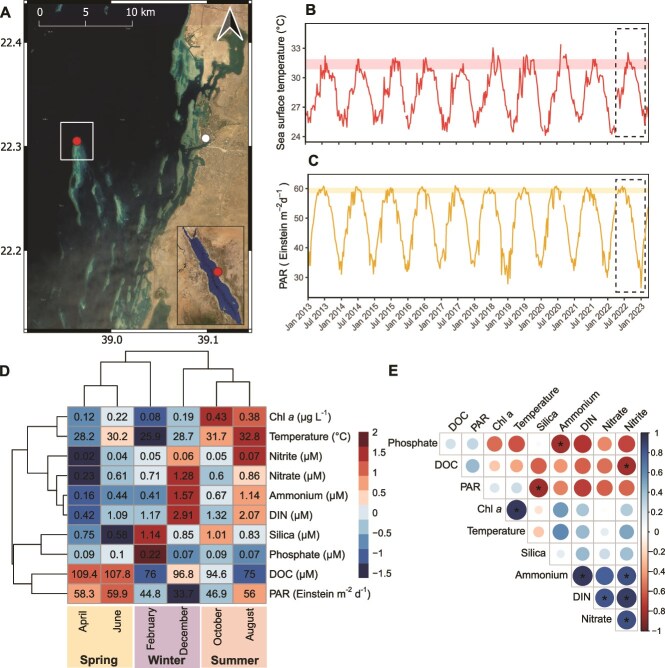
Natural environmental variation at the sampling site. **(A**) Sampling site (red dot) in the Central Red Sea, close to the King Abdullah University of Science and Technology (KAUST, white dot). **(B**) Sea surface temperature and **(C**) photosynthetically available radiation (PAR) of the past ten years, extracted for the reef area (white box in **A**) using GIOVANNI, NASA. Dashed boxes indicate the study period (April 2022 – February 2023) and red and yellow horizontal bars indicate the mean ± sd range of SST in summer 2022, and PAR in spring 2022, respectively. (**D**) Hierarchal clustering heatmap of z-score normalized environmental parameters. Values in cells represent mean values per month and colors of cells represent Z-scores. (**E**) Spearman correlation plot of environmental variables. Colors represent R^2^ values. Significant (*P* < .05) correlations are marked with asterisks. Chl *a* = chlorophyll *a*, DIN = dissolved inorganic nitrogen, DOC = dissolved organic carbon.

Sea surface temperatures (SSTs) and photosynthetic radiation (PAR) data was extracted from an area-averaged time series produced with the Giovanni online data system, developed and maintained by the NASA GES DISC. We used the MODIC-Aqua (8-daily), 4 km datasets for SST (11 μm, daytime), and photosynthetically available radiation (PAR, R. Frouin) [35]. All values were averaged for an area with the study site located in its center (38.94798E, 22.28716 N, 38.98138E, 22.32320 N; white box in Fig. 1A). Temperatures in summer 2022 were comparable with the previous years (see red horizontal box in Fig. 1B) and PAR was always highest in spring (see yellow horizontal box in Fig. 1C). SST data was further validated by in situ temperature measurements obtained with a temperature logger (Onset Hobo pendant; sampling interval 10 min) deployed at the sampling site at 1–2 m depth for the entire duration of the experiment (see Supplementary Fig. S2).

The three “seasons” are based on the hierarchal clustering of months according to similarities in environmental data (Fig. 1D). The spring months April and June were characterized by low inorganic nutrient concentrations (i.e. DIN forms, Phosphate and Silica), increasing temperatures, and highest PAR and dissolved organic carbon (DOC) concentrations. The summer months August and October exhibited the highest temperatures and chlorophyll *a* concentration, and intermediate nutrient concentrations and PAR. The winter months December and February were characterized by peaks in inorganic nutrient concentrations, decreasing temperatures, and the lowest PAR. Overall, water chlorophyll *a* concentration correlated positively with temperature, and nitrate and ammonium concentrations correlated with each other (and with DIN, i.e. the sum of nitrate, nitrite, and ammonium, Fig. 1E). Negative correlations were observed between PAR and silica, phosphate and ammonium, and DOC and nitrite.

### Stable isotope analyses

For C and N elemental and stable isotope ratio analyses, coral fragments of approximately 5 cm length were defrosted on ice for 30 min. 5 ml of MilliQ water was filled into an airbrush, attached to a high-pressure air valve. Coral fragments were placed into a sterile transparent plastic bag and tissue was blasted off with the airbrush. Tissue slurry was transferred into a sterile, labelled falcon tube and stored at −20°C until further processing. Details on separation of host tissue from Symbiodiniaceae cells for stable isotope analysis are presented in the supplementary material (Supplementary Text S3).

Analyses of C and N elemental and stable isotope ratios were performed as previously described by Rix et al. [36] on host tissue and Symbiodiniaceae separately. Isotope ratios (r) are the heavier: lighter isotope (^13^C:^12^C, ^15^N:^14^N), standardized to reference material of Vienna Pee Dee Belemnite for δ^13^C (0.01118) and atmospheric N for δ^15^N (0.00368), according to the formula: δX = (r_sample_/r_reference_ − 1) ^*^ 1000, given as δ^13^C or δ^15^N (‰).

### DNA extraction and sequencing

DNA extraction was performed on 1 cm coral fragments from the −80°C freezer (see “sample collection”) using the DNeasy Blood and Tissue Kit (Qiagen) following the protocol for total DNA purification from animal tissues, with some adaptations (see Supplementary Text S4). The DNA concentration per sample was quantified using a Qubit dsDNA assay kit (Invitrogen) and DNA quality was checked using a Nanodrop 8000 Spectrophotometer. PCR amplification of the V3-V4 region of the bacterial 16S rRNA gene was performed using the universal primers 341F (5′-CCTACGGGNGGCWGCAG-3′) and 805R (5′ GACTACHVGGGTATCTAATCC-3′). After verification of the presence of PCR products by gel electrophoresis, indexed PCR was performed followed by normalization and equimolar pooling. Sequencing was conducted on the MiSeq platform (MiSeqFGx, Illumina) with v3 chemistry.

### Sequence data processing

Demultiplexed raw sequence reads were imported to R version 4.2.2 [37] for processing using the Dada2 package [38] (see details in Supplementary Text S5). The final ASV table, sample metadata, and taxonomic assignments were imported to *Phyloseq* [39] for downstream processing. Within *Phyloseq*, all non-bacterial reads, chloroplasts, or mitochondria were removed based on their taxonomic assignments. ASV tables were then permutationally rarefied to 2900 reads per sample using the *rrarefy.perm()* function of the *EcolUtils* package with 1000 permutations [40]. Following Schloss [41], we decided to settle for this relatively low sampling depth, as it (i) still captured the diversity of most samples (Supplementary Fig. S6), (ii) maximized the number of samples that could be retained, and (iii) the loss of rare taxa was deemed to be of less importance since the focus was on the functional potential of the bacterial communities.

To assess differences in the functional potential of the host-associated microbiomes across coral species and sampling time points, we used the standalone *PICRUSt2* software [42] with its default settings on the rarefied ASV table. This software uses taxonomic annotations of 16S rRNA sequences to predict the metabolic functional pathways present in bacterial communities. We used the default cutoff of Nearest Sequence Taxon Index (NSTI) of 2, and remaining ASVs (99.9% < 2) had a mean of 0.217 ± 0.235 and a range of 0.000–1.963. The MetaCyc database [43] was further used to annotate pathway IDs from *PICRUSt2* and merge them into pathway groups. We acknowledge that using 16S rRNA data to infer metabolic functions comes with limitations: (i) The accuracy of the predictions depends on the quality and size of the reference database and may be limited for some taxa and pathways [42, 44], and (ii) as identification to the microbial strain level is not possible, pathways specific to certain strains are not included [42].

### Statistical analyses

**Figure 2:**
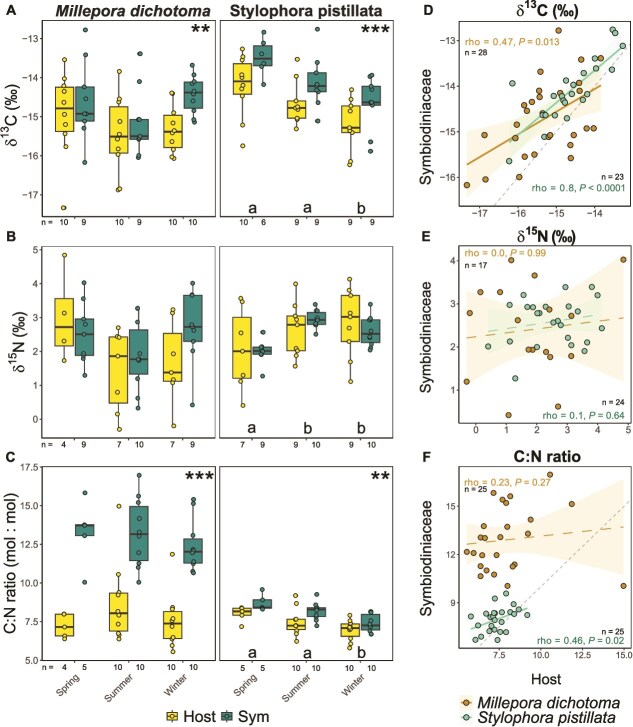
Carbon (C) and nitrogen (N) stable isotope- and elemental ratios of *M. dichotoma* and *S. pistillata* host and Symbiodiniaceae compartment per season (spring: April and June 2023; summer: August and October 2023; winter December 2024 and February 2024) (**A–C**) and correlated for Symbiodiniaceae vs. host tissue across all months (**D–F**). April data was excluded for C:N ratios due to insufficient sample material. Asterisks in **A–C** indicate significant difference between holobiont compartments (^**^  *P* < .01, ^***^  *P* < .001) and lower case letters indicate significant differences between seasons *(*fdr-adjusted *P* < 0.05). Spearman’s rho and *P*-value are plotted within each correlation plot (**D–F**) and a solid best fit line indicates a significant correlation. Grey dashed lines indicate the 1:1 line/identity line.

**Figure 3:**
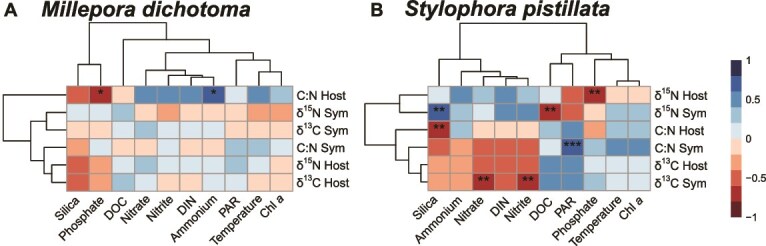
Spearman correlation matrices of host- and Symbiodiniaceae (Sym) stable isotope- and C:N ratios with environmental variables for both coral species. Asterisks indicate significant spearman correlations (fdr-corrected ^*^  *P* < .05, ^**^  *P* < .01, ^***^  *P* < .001; *R^2^* > .6). Colour gradient and heatmap clustering is based on spearman correlation coefficients, with blue indicating positive- and red negative associations.

All statistical analyses were conducted with R Studio (version 2024.09.1, R version 4.4.1). Stable isotope and C:N ratio data (Figs 2A–C) were analysed using linear mixed-effects models (*lmer* package) after assumption testing (*performance* package) and outlier removal (*rstatix* package). Correlations between tissue compartments (Figs 2D–F) and with environmental parameters (Fig. 3) were tested using Spearman correlation tests. Alpha levels were set to *P* = .05 after correction for false discovery rate (fdr).

**Figure 4:**
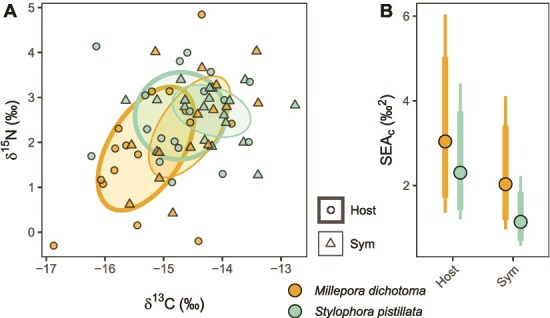
Stable isotope niches of *M. dichotoma* and *S. pistillata*. (**A**) Standard ellipse areas (40% core isotopic niche) for coral host- and Symbiodiniaceae (Sym) compartments of both species. (**B**) Bayesian standard ellipse areas corrected for sample size (SEAc) with mode (point) and credible intervals (thick line = 50%; thin line = 95%).

For stable isotope niche analyses, we only used fully paired samples (i.e. complete sets of δ^13^C and δ^15^N values for both tissue fractions) to avoid potential bias. We plotted standard ellipse areas (central 40% probability ellipse) for coral host- and Symbiodiniaceae (Sym) tissues of both species to visualize their isotopic niches (Fig. 4A). For comparison of niche sizes (Fig. 4B), we calculated Bayesian standard ellipse areas (SEA_b_) using the *SIBER* package [45] (*siberMVN* and *siberEllipses* functions) and corrected for small sample sizes of each group (*n* = 16 for *M. dichotoma*; *n* = 18 for *S. pistillata*) to obtain the standard ellipse area corrected for sample size (SEA_c_) by multiplying with the group-specific factor ((n − 1)/(n-2)) [45]. We further calculated the species- and tissue-type specific Layman metrics [46] (*laymanMetrics* function of *SIBER*).

**Figure 5:**
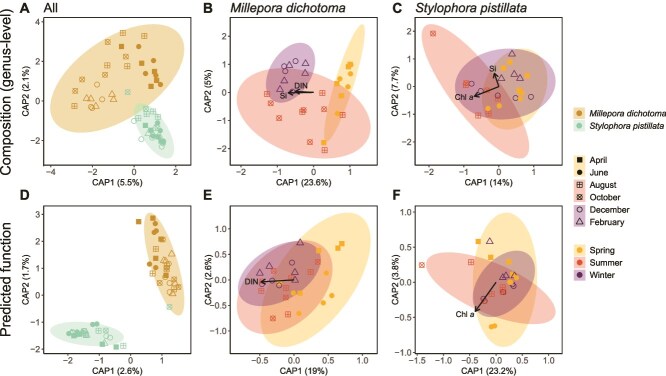
Canonical analysis of principal coordinates (CAP) of the community structure at the genus-level (**A–C**) and predicted metabolic functions (**D–F**) of the bacterial communities associated with the corals *M. dichotoma* and *S. pistillata*. Significant (*P* > .05) environmental predictors are plotted as vectors. Percent values on axes represent the amount of variation explained by the first two dimensions. Bray-Curtis dissimilarities were calculated on square root-transformed data to estimate differences between samples. Si = silica; DIN = dissolved inorganic nitrogen; DOC = dissolved organic carbon; PO4 = phosphate; spring = April and June 2023; summer = august and October 2023; winter = December 2024 and February 2024.

**Figure 6:**
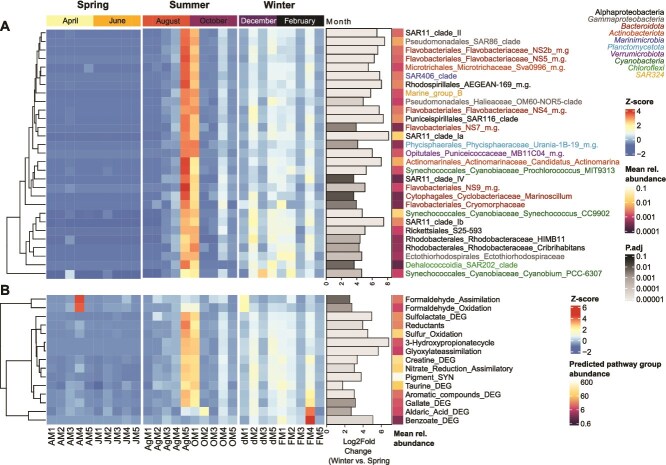
**(A)** Bacterial taxa (genus-level) associated with *Millepora dichotoma* significantly increased relative abundance in winter compared to spring (summer did not significantly differ from winter and/or spring as determined via PERMANOVA, see table, and was therefore not considered in compositional and pathway enrichment analyses). **(B**) Predicted pathway groups significantly enriched in winter compared to spring in *M. dichotoma*. Note that no significant overall change in predicted metabolic functions was observed between seasons (see table 3). Gray bars indicate a significant difference in winter compared to spring months (DESeq2 adjusted *P* < .01, absolute Log2FoldChange ≥ 1.5). M.G. = marine group; DEG = degradation; SYN = biosynthesis; spring: April and June 2023; summer: August and October 2023; winter December 2024 and February 2024.

Differences in bacterial community composition and function between groups (Fig. 5) were assessed using two-way multivariate analysis of variance (PERMANOVA, *vegan* package). Differential expression analysis (DESeq2 *package*) was used to assess seasonal differences in *M. dichotoma* (Fig. 6). For further details on statistical analyses, assumption testing, and post-hoc tests, see Supplementary Text S7.

## Results

### Carbon and nitrogen stable isotopes and elemental ratios in coral host and Symbiodiniaceae

The δ^13^C values in *M. dichotoma* were − 15.2 ± 0.9‰ for host- and − 14.7 ± 0.8‰ for Symbiodiniaceae (always mean ± standard deviation) and differed significantly between holobiont compartments (i.e. coral host and Symbiodiniaceae), but not between seasons (Fig. 2A, Table 1). There was a weak but significant correlation (*rho* = 0.47; *P* = .013) between host- and Symbiodiniaceae δ^13^C values (Fig. 2D). The δ^15^N values in *M. dichotoma* were 1.9 ± 1.3‰ for host- and 2.4 ± 1.0‰ for Symbiodiniaceae and were not significantly different between holobiont compartments nor between seasons (Fig. 2B, Table 1), with no correlation between holobiont compartments (Fig. 2E). The molar C:N ratios in *M. dichotoma* were significantly enriched in Symbiodiniaceae (12.9 ± 1.9) relative to host tissue (8.0 ± 2.1) by 61% and neither a significant season effects (Fig. 2C, Table 1) nor correlation of C:N ratios between holobiont compartments (Fig. 2F) were observed. Spearman correlation analyses of host- and Symbiodiniaceae tissue variables with environmental parameters only revealed weak correlations (fdr-adjusted *P* < .05) of host C:N ratios with phosphate (negative) and Ammonium (positive) (Fig. 3A).

**Table 1:** Results of linear mixed-effects models (Type III ANOVA table with Satterthwaite’s method) for fixed effects of season (Spring: April and June 2023; Summer: August and October 2023; Winter: December 2024 and February 2024) and holobiont compartments (coral host or Symbiodiniaceae) and random effects of Colony ID on C and N stable isotope- and elemental ratios of *M. dichotoma* and *S. pistillata*. *P*-values are adjusted to control for false discovery rate (*fdr*) across all tests. Significant effects are marked in bold (*P.adj* < .05). NumDF = numerator degrees of freedom, DenDF = Denominator degrees of freedom.

	δ^13^C				δ^15^N				C:N			
	*NumDF/DenDF*	*F*	*P*	*P.adj*	*NumDF/DenDF*	*F*	*P*	*P.adj*	*NumDF/DenDF*	*F*	*P*	*P.adj*
*M. dichotoma*												
Season	2/26	1.5	.25	.44	2/26	3.3	.054	.12	2/22	1.2	.24	.44
Tissue	1/25	12.1	.002	.008	1/24	1.1	.31	.44	1/22	80.4	< .001	< .001
Season ^*^ Tissue	2/25	1.2	.32	.44	2/24	1.7	.20	.40	2/22	0.5	.58	.69
*S. pistillata*												
Season	2/25	5.5	.01	.03	2/48	5.7	.006	.02	2/22	10.6	< .001	.004
Tissue	1/20	29	< .001	< .001	1/48	0.0	.95	.95	1/22	11.1	.003	.01
Season ^*^ Tissue	2/19	0.4	.65	.73	2/48	0.7	.48	.62	2/22	0.3	.77	.82

The δ^13^C values of *S. pistillata* were significantly affected by holobiont compartments and season (Fig. 2A, Table 1). Symbiodiniaceae δ^13^C values were always higher by 0.6 ± 0.1‰ (overall mean = −14.2 ± 0.8‰) relative to host tissues (overall mean =−14.7 ± 0.8‰). δ^13^C values of *S. pistillata* were significantly higher in spring compared to winter by 1.1 ± 0.1‰ (Fig. 2A). The correlation between host- and Symbiodiniaceae δ^13^C values was strong and highly significant (*rho* = 0.80; *P* < .0001) (Fig. 2D). *S. pistillata* δ^15^N values were 2.5 ± 1.0‰ for host- and 2.5 ± 0.5‰ for Symbiodiniaceae with no significant difference between holobiont compartments but a significant season effect. The δ^15^N values of *S. pistillata* were significantly enriched in summer and winter compared to spring by 0.7 ± 0.1‰ (Fig. 2B, Table 1). C:N ratios of *S. pistillata* were significantly affected by holobiont compartments and season (Fig. 2C, Table 1).

Symbiodiniaceae C:N ratios of *S. pistillata* were consistently higher by an average of 0.71 ± 0.2 units (overall mean = 8.0 ± 0.8) relative to host tissue (overall mean = 7.4 ± 0.8) and were significantly reduced in winter relative to spring by 14% and in winter relative to summer by 9% (see letters in Fig. 2C). The correlation between host- and Symbiodiniaceae C:N ratios was weak but significant (*rho* = 0.46; *P* = .021) (Fig. 2F). Spearman correlation analyses of host- and Symbiodiniaceae tissue variables of *S. pistillata* with environmental parameters revealed five significant negative- and two significant positive correlations (fdr-adjusted *P* < .01, Fig. 3B). Most notably, C:N ratios in Symbiodiniaceae correlated positively with PAR (*P* < .001), while δ^15^N in host and Symbiodiniaceae correlated negatively with phosphate (*P* < .01) and DOC (*P* < .01), respectively. δ^13^C values in Symbiodiniaceae were negatively correlated with nitrate and nitrite concentrations.

### Stable isotope Bayesian niche analysis

Bayesian analysis of isotopic niches revealed similar niche sizes (Bayesian standard ellipse area corrected for sample size, SEA*_c_*) for host tissue of the two coral species. The niche size of *S. pistillata* Symbiodiniaceae tissue was however significantly smaller compared host tissue of *M. dichotoma* and *S. pistillata* by 63% and 50%, respectively (Fig. 4A and B; > 98% posterior probability of group difference)*.* This trend was also confirmed by the Layman metrics (Table 2). Specifically, Symbiodiniaceae of *S. pistillata* displayed the lowest δ^15^N range (39% below mean of all groups), total area (44% below mean), distance to centroid (31% below mean) and nearest neighbour distance (NND, 27% below mean).

**Table 2:** Layman metrics [46] of stable isotope data for each species and holobiont compartments (Host = coral host; Sym = Symbiodiniaceae). Bold indicates the highest, and italic the lowest values out of the four groups. Descriptions are adapted to relate to samples within one group instead of species within a community.

		*M. dichotoma*		*S. pistillata*	
Metric	Description (adapted from Layman et al. 2007)	Host	Sym	Host	Sym
δ^15^N range	Larger δ^15^N range suggests a greater degree of trophic diversity.	**5.144**	3.608	3.025	*2.121*
δ^13^C range	Larger δ^13^C range suggests a greater degree of trophic diversity with varying C sources.	**3.032**	2.190	*2.700*	2.885
Total Area	Convex hull area (including all samples) in the δ^13^C - δ^15^N biplot space. Proxy of the extent of trophic diversity.	**8.011**	5.504	6.711	*3.298*
Distance to centroid	Mean Euclidean distance of samples to the δ^13^C - δ^15^N biplot centroid. Indicates the average degree of trophic diversity.	**1.281**	1.118	1.075	*0.729*
Nearest neighbour distance (NND)	Mean Euclidean distance to the nearest neighbour in the δ^13^C - δ^15^N biplot space. Measure of density. Low value indicates low divergence of samples.	**0.557**	0.459	0.530	*0.343*
Standard deviation of NND	Measure of the evenness of spacing between samples. Low SDNND indicates more even distribution.	**0.526**	0.281	0.338	*0.263*

### Coral-associated bacterial community composition and functional predictions

The bacterial communities associated with *M. dichotoma* and *S. pistillata* differed significantly from each other throughout the study and explained 46% of the overall variation (PERMANOVA species effect, Fig. 5A, Supplementary Table S8, Fig. S9). The bacterial community of *S. pistillata* was dominated (82 ± 11%) by the genus *Endozoicomonas* throughout the study, which only represented 4% of the bacterial community in *M. dichotoma* (Supplementary Fig. S9). The most abundant genus in *M. dichotoma* was *Spirochaeta* (27 ± 15%) (Supplementary Fig. S9), which was rare in *S. pistillata* (0.5 ± 0.3%). The bacterial community of *M. dichotoma* had a significantly higher alpha diversity (Shannon diversity) compared to *S. pistillata* (2.5 ± 0.7 vs. 0.9 ± 0.4, respectively; ANOVA: *F*_(1,52)_ = 113, η^2^_G_ = 0.69, *P* < .001). Beta diversity (Bray–Curtis dissimilarity) was also significantly higher in *M. dichotoma* compared to *S. pistillata* (PERMANOVA: *F* = 87, *R^2^* = 0.63, *P* = .001; Fig. 5A). Predicted metabolic functions were significantly different between the two coral species, explaining 72% of the variation (PERMANOVA species effect, Fig. 5D, Supplementary Table S8).

The interaction of species and season was significant and explained 6% of the overall variation in bacterial community compositions (Supplementary Table S8). When testing each species individually, season had a significant effect for *M. dichotoma* but not for *S. pistillata* (Fig. 5B and C, Supplementary Table S8). Pairwise PERMANOVAs revealed that bacterial community compositions of *M. dichotoma* differed significantly between spring (April and June) and winter (December and February; Fig. 5B, Supplementary Table S8). Distance-based redundancy analysis (dbRDA) further revealed that environmental concentrations of Silica, and DIN significantly correlated with bacterial community composition of *M. dichotoma*, parallel to the CAP1 axis which explained 24% of the variation and separated the seasons spring and winter (Fig. 5B). Seasons had no significant overall effect on predicted metabolic functions in any of the bacterial communities associated with the two coral species (Figs 5E–F, Supplementary Table S8).

### Temporal change in the bacterial communities associated with *M. dichotoma*

Due to the significant seasonal differences in the bacterial communities associated with *M. dichotoma* in spring versus winter (Supplementary Table S8), we further conducted a differential abundance analysis to compare genus-level relative abundances between these two seasons. A total of 29 bacterial genera were significantly different in spring versus winter in *M. dichotoma*, and all genera had higher relative abundances in winter compared to spring (Fig. 6A). Out of these 29 genera, the most abundant were *Synechococcus* CC9902 (0.2 ± 0.3% in spring vs. 5.0 ± 2.3% in winter) and SAR11 clade 1a (0.02 ± 0.03% in spring vs. 3.6 ± 1.5% in winter).

Although there was no overall seasonal effect on predicted metabolic functions (Supplementary Table S8), we conducted a differential abundance analysis to explore the functional implications of the observed significant shift in bacterial community composition between spring and winter. The analysis revealed that 15 groups of predicted metabolic functions were significantly enriched in winter versus spring (Fig. 6B). These predicted functional groups were related to C metabolism (3-hydroxypropionatecycle; glyoxylate assimilation; Reductants; formaldehyde pathways), N cycling (assimilatory nitrate reduction; creatine degradation), S compound degradation (taurine degradation; sulfolactate degradation; S oxidation), degradation of complex organic molecules (i.e. aromatic compounds, gallate, aldaric acid, benzoate), and pigment synthesis.

## Discussion

In this study, we show distinct differences in the internal C/N cycling and dynamics in associated bacterial communities between the scleractinian coral *S. pistillata* and the hydrocoral *M. dichotoma*. These results suggest a fundamental difference in how these coral holobionts adapted strategies for maintaining (i.e. regulating) or shifting (i.e. conforming) key physiological traits during seasonal changes in environmental conditions.

### Stable C/N cycling and bacterial community composition suggest a regulator-like life history strategy in *S. pistillata*

The correlations in δ^13^C values (Fig. 2D) as well as the C:N ratios (Fig. 2C) between the coral-host and Symbiodiniaceae indicate a tight C cycling within the *S. pistillata* holobiont [33, 47, 48]. Interestingly, this correlation was significant despite sampling over a 12-month period characterized by pronounced seasonal variation in environmental parameters (Fig. 1D), suggesting stable C/N cycling through regulation of internal conditions by the coral holobiont (*sensu* [22]). Both host and Symbiodiniaceae δ^13^C values were lower by 1.1‰ in winter compared to spring (Fig. 2A). This lower value can be explained by a higher light availability in spring (Fig. 3B), which stimulated photosynthesis, resulting in reduced discrimination against the heavier ^13^C isotope due to increasing inorganic carbon limitation [49–51]. An increased DIN availability (Fig. 1D) may have further contributed to the depleted δ^13^C values in winter [52]. Although there was no correlation of δ^15^N values between coral host and Symbiodiniaceae (Fig. 2E), the similarities in δ^15^N values in both holobiont compartments indicate a shared N source [53] and/or tight N recycling within the holobiont [54]. In fact, the δ^15^N values between 0 and 4‰ may suggest diazotroph-derived N as a main N source [55–57], as previously described for *S. pistillata* from the northern Red Sea [58]. Values at the higher end of this δ^15^N range could also be influenced by heterotrophic feeding on POM which can have δ^15^N values of about 4 to 6‰ in the Central Red Sea [59]. The low δ^15^N range in *S. pistillata* Symbiodiniaceae (39% below the mean of all groups, Table 2) also resulted in a small isotopic niche size (50%–63% smaller compared to host compartments) (Fig. 4A and B). A small isotopic niche has been previously reported for *S. pistillata* from Taiwan and can be interpreted as restricted resource use and/or regulation of internal conditions by the coral host [60]. Overall, our analyses consistently support a tight coupling in C/N cycling in the *S. pistillata* holobiont and a stable symbiosis despite pronounced environmental variations facilitated by the regulation of Symbiodiniaceae within the coral host.

Additionally, bacterial communities within *S. pistillata* remained stable throughout the study period (Fig. 5A and C). Members of the genus *Endozoicomonas* were consistently dominant (59%–96% relative abundance), corroborating previous studies in the Red Sea [32, 61] and beyond [62]. This bacterial genus is a common symbiont of marine species, and although its functions for host organisms are not well understood, they can likely be associated with C and N cycling dynamics (reviewed by [63]). A similar stability in the bacterial community of *S. pistillata* despite pronounced variations in environmental parameters was also observed in manipulative long-term heat stress experiments in the northern Red Sea [54]. Interestingly, Savary et al. [54] further showed a fast and pervasive change in gene expression of the coral host as well as the Symbiodiniaceae during heat stress, with a rapid recovery to baseline levels once the stress was alleviated. This so-called transcriptomic resilience may therefore further indicate the tight control *S. pistillata* exerts on its associated bacterial community, underpinning its regulator-like life history strategy.

### Flexible C/N cycling and bacterial community composition suggest a conformer-like life history strategy in *M. dichotoma*

In contrast to *S. pistillata*, the correlation between *M. dichotoma* host and Symbiodiniaceae tissue was found to be weak for δ^13^C values (Fig. 2D) and not significant for C:N ratios (Fig. 2F), indicating a more flexible C cycling within the holobiont [22, 47, 48]. This flexibility in nutrient cycling is also supported by a considerably higher stable isotope niche size in *M. dichotoma* Symbiodiniaceae tissues compared to symbionts of *S. pistillata* (44% larger SEA_c_ with >95% probability, Fig. 4). Both δ^13^C and δ^15^N ratios were within similar ranges for coral host and Symbiodiniaceae, suggesting shared nutrient sources and/or recycling of C and N [53]. Elevated C:N ratios of Symbiodiniaceae (i.e. 12.9 ± 2.0) compared to host tissue (i.e. 7.9 ± 2.1) may however indicate a certain decoupling of the two-way nutrient exchange, either by the restriction of N supply from the host and subsequent N-limitation in the Symbiodiniaceae [23, 64, 65] and/or the retention of the photosynthetically fixed C within the Symbiodiniaceae [66]. Both δ^13^C and δ^15^N values of the hydrocoral host and Symbiodiniaceae revealed a high intra-seasonal variation (Fig. 2A and B), with the host tissues displaying the largest trophic niche (Fig. 4A and B), with the highest δ^15^N range (Table 2). This high variation in parameters suggests a considerable intraspecific variation in C/N cycling in *M. dichotoma* holobionts with potentially higher trophic diversity compared to *S. pistillata* [67]. Since no temporal effect was observed, the variation in δ^13^C and δ^15^N values was likely not driven by seasonal changes in environmental parameters, but rather by (i) genotype, (ii) microenvironments on the reef [66], and/or (iii) differences in the associated microbial communities among individual colonies [68, 69].

The bacterial community composition was indeed more diverse and variable among *M. dichotoma* colonies compared to *S. pistillata* (Fig. 5A). Members of the genus *Spirochaeta* were the most dominant taxa (5%–55% relative abundance) corroborating other studies performed in the Red Sea [28, 29]. This ubiquitous presence may point towards functional importance [70, 71], such as the provision of photosynthetically fixed C and/or fixed N to the host, as suggested for Mediterranean octocorals [72] and termites [73, 74]. Future studies should consider investigating the functional roles of *Spirochaeta* affecting the physiology and nutrition of *Millepora* hydrocorals. In contrast to C/N cycling, the bacterial community composition showed a clear temporal pattern (Supplementary Fig. S9), with 29 bacterial genera significantly increasing in relative abundance in winter compared to spring, which combined made up 20 ± 9% in winter and < 1% in spring (Fig. 5A). The six bacterial taxa that contributed the most to the overall bacterial community in winter (i.e. *Synechococcus*, SAR11 clade 1a & 1b, *Candidatus Actinomarina*, SAR86 clade, SAR116 clade; all >1% mean relative abundance in winter) are common members of the bacterioplankton community of the Red Sea [75, 76]. Therefore, these can be likely associated with an increased influx of bacterioplankton from the surrounding seawater into the coral holobiont. Future studies should consider assessing the seasonal changes of reef bacterioplankton to investigate if seasonal changes in *M. dichotoma* microbiomes are associated with these variations in the water column bacterioplankton communities.

Although variations in bacterial community composition did not result in an overall change of the predicted functions (Fig. 5E), 15 predicted functional groups were found to increase in winter relative to spring (Fig. 6B). These functional groups were predominantly associated with C, N, and S cycling, potentially contributing to the hydrocoral holobiont’s capacity to respond to environmental fluctuations to adapt to changing environmental conditions [29]. For instance, enhanced C and N provisioning by *M. dichotoma*-associated bacteria may help mitigate seasonal constraints in light and nutrient availability during winter. It is important to note that these predictions represent the functional potential of the microbial community and do not allow us to directly derive the activity of these pathways and/or quantify metabolic rates (see Materials and Methods section for further limitations). The fact that neither δ^13^C (Fig. 3A) nor δ^15^N values (Fig. 3B) showed a significant seasonal variation in both holobiont compartments indicates that these changes in the functional profile of the associated bacterial community may have only had a minor contribution to the holobiont C/N cycling. Overall, a high intra-seasonal variation in δ^13^C and δ^15^N values, a large trophic niche and spread, as well as temporal variability in the associated microbes point towards a more pronounced influence of environmental parameters on holobiont functioning, thus supporting a conformer-like life history strategy of *M. dichotoma*.

### Ecological implications of different life-history strategies

Organisms can achieve a wide physiological tolerance to fluctuations in environmental parameters by employing two contrasting adaptive strategies: either maintaining internal homeostasis (as observed in *S. pistillata*) or aligning their internal states with changing external conditions (as seen in *M. dichotoma*). These contrasting adaptation strategies, often referred to as “regulator” and “conformer” life history-strategies, likely arise from evolutionary trade-offs, including energetic costs, environmental variability, and performance, yet these variations in adaptive strategies are not fully understood (e.g. [77]).

Regulators, which maintain stable symbiotic relationships and internal conditions, may experience limitations in their ecological and trophic niche [78]. However, this internal stability often provides increased resistance to high variability in environmental conditions, such as heat waves and other stress events [79]. In contrast, conformers exhibit greater flexibility, allowing their internal environment and associated microbial communities to shift in response to external changes. This flexibility can increase susceptibility to pathogens due to less stable bacterial communities [80], but may concomitantly enhance trophic plasticity by enabling the holobiont to supplement its metabolism through additional C and N pathways or vitamin synthesis [7, 8, 79]. Such adaptability may be particularly beneficial when environmental disturbances are infrequent.

It should be noted that this strict dichotomy between regulators and conformers is likely an oversimplification. Many organisms exhibit a combination of both strategies, depending on the environmental parameter. For instance, many fish species act as temperature-conformers but ion-regulators [81]. Furthermore, by unifying physiological and ecological concepts, Meunier et al. [82] proposed that conformers do not regulate for intermediate conditions of a given parameter but begin to regulate once environmental extremes are reached, whereas regulators actively maintain internal homeostasis within their comfort zone but may shift to a conformer-like strategy beyond critical thresholds.

This study reveals distinct differences in the internal C/N cycling and bacterial community dynamics between the conformer *M. dichotoma* and the regulator *S. pistillata* in response to seasonal environmental changes. These distinct life-history strategies, combined with their historically narrow environmental ranges, make *M. dichotoma* and *S. pistillata* valuable models for exploring the boundaries of conformer and regulator concepts. Identifying the specific environmental “breaking points” will be crucial for predicting the future resilience and adaptability of coral holobionts under climate change scenarios.

## Supplementary Material

ESM_Thobor_et_al_resubmission_ycag008

## Data Availability

The data and R codes are available on Zenodo data repository (https://doi.org/10.5281/zenodo.15189718) and the entire Raw sequence reads of 16S rRNA amplicon data is deposited in NCBI under the Bioproject PRJNA1248719. Moreover, isotopic raw data as well as data clean up procedure including two additional coral species are available at Zenodo (https://doi.org/10.5281/zenodo.17849457).
