# Supplementary material for: Contrasting physiological adaptation strategies to natural environmental change in two Red Sea coral holobionts
Source: ISME Commun. 2026 Feb 6;6(1):ycag008. doi: 10.1093/ismeco/ycag008 (PMC12903954; doi:10.1093/ismeco/ycag008)
Supplement: ESM_Thobor_et_al_resubmission_ycag008 [file esm_thobor_et_al_resubmission_ycag008.pdf]

Electronic Supplementary Materials

Contrasting physiological adaptation strategies to natural environmental change in two Red Sea coral holobionts

Bianca M. Thobor[1], Claudia Hill[1,2], Gordon F. Custer[3,4,5], Neus Garcias-Bonet[2], Michael D. Fox[2], Yusuf C. El-Khaled[2], Eva Aylagas[2], Francisco Dini-Andreote[3,4], Ulrich Struck[6,7], Arjen Tilstra[*1,8], Raquel Peixoto[2], Susana Carvalho[*2], Christian Wild[1], Benjamin Mueller[*1,9]

[1] Department of Marine Ecology, University of Bremen, Leobener Strasse 6, 28359 Bremen, Germany

[2] Biological and Environmental Sciences and Engineering (BESE) Division, King Abdullah University of Science and Technology (KAUST), Thuwal 23955-6900, Saudi Arabia

[3] Department of Plant Science and Huck Institutes of the Life Sciences, The Pennsylvania State University, University Park, PA 16802, USA

[4] The One Health Microbiome Center, Huck Institutes of the Life Sciences, The Pennsylvania State University, University Park, PA 16802, USA

[5] C., University of Maryland Eastern Shore, MD 21853, USA

[6] Museum für Naturkunde, Leibnitz Institute for Evolution and Biodiversity Science, Invalidenstrasse 43, 10115 Berlin, Germany

[7] Department of Earth Science, Free University Berlin, Malteserstrasse 74-100, 12249 Berlin, Germany

[8] Arcadis Nederland B.V., Amsterdamseweg 13, 6814 CM Arnhem, The Netherlands

[9] Department of Oceanography and Sea Grant College Program, Center for Microbial Oceanography: Research and Education, University of Hawai'i at Mānoa, 1000 Pope Road, Honolulu, HI 96822, USA

* Corresponding authors:

Benjamin Mueller – muellerb@ymail.com ; Department of Marine Ecology, University of Bremen, Leobener Strasse 6, 28359 Bremen, Germany

Susana Carvalho – susanna.carvalho@kaust.edu.sa ; Biological and Environmental Sciences and Engineering (BESE) Division, King Abdullah University of Science and Technology (KAUST), Thuwal 23955-6900, Saudi Arabia

Arjen Tilstra – arjentilstra@gmail.com ; Arcadis Nederland B.V., Amsterdamseweg 13, 6814 CM Arnhem, The Netherlands

Table of Content:

Text S1: Processing and analysis of environmental chlorophyll and nutrient concentrations 03

Figure S2: *In situ* water temperature during the experimental period 06

Text S3: Separation of host tissue from Symbiodiniaceae cells for stable isotope analyses 07

Text S4: Adapted DNA extraction using DNeasy Blood and Tissue Kit 08

Text S5: Processing of demultiplexed raw sequence reads using the Dada2 package 09

Figure S6: Refractory curve of bacterial taxa associated with *M. dichotoma* and *S. pistillata* 10

Text S7: Standard statistical analyses 11

Table S8: PERMANOVA results of species and season effects for bacterial community compositions and metabolic functions 13

Figure S9: Bacterial taxa associated with *M. dichotoma* and *S. pistillata* 14

Figure S10: Representative pictures of the studied species taken at the sampling site. 15

ESM Text S1: Processing and analysis of environmental chlorophyll and nutrient concentrations

On-board sample processing. Nitrate, nitrite, phosphate, and silicate samples (6 x 15 mL) were passed over a 0.22 µm syringe filter (Millex®-GV) into 15 mL cone-shaped sample tubes. Ammonium samples were directly filled into 15 mL cone-shaped sample tubes. Unfiltered aliquots for dissolved organic carbon (DOC; 500 mL) and chlorophyll *a* (4 L) were transferred into acid-washed (4% HCl) vessels and two opaque bottles, respectively. All aliquots were kept in the dark and on ice during the transport back to KAUST and subsequently stored at 4 °C (DOC and chlorophyll *a*) or -20 °C (inorganic nutrients) for further analysis.

Environmental chlorophyll. Aliquots for chlorophyll *a* were filtered over GF/F filters (0.7 µm nominal pore size), desalinated by flushing with 5 mL ultrapure water before being stored at -80 °C for further processing. Chlorophyll *a* concentrations were determined by placing each filter in a cone-shaped sample tube (15 mL) and immersed in 10 mL of 90% acetone. Tubes were then vortexed for 1 min, sonicated for 10 min in an ice bath, and vortexed again for 30 min. Tubes were subsequently covered with aluminium foil to prevent light exposure and stored at 4 °C overnight. The next day, the vortexing and sonication steps were repeated two more times, followed by centrifugation (10 min at 1211 x g and 4 °C). After samples were brought to room temperature, a 2 mL aliquot was transferred into a glass cuvette (path length = 12 mm), and the extinction at $\lambda = 664$ nm was measured on a fluorometer (Turner Design Trilogy) before and after addition of 100 µL of 5% HCl, per sample. The difference between these readings provided the chlorophyll *a* concentration. Before and after every sample a blank of 90% acetone and a chlorophyll *a* standard (Sigma Aldrich) were measured as the negative and positive control, respectively.

Dissolved organic carbon. Aliquots of water samples were processed within one day of collection by passing through a pre-combusted (4.5h at 450 °C) GF/F filter (0.7 µm nominal pore size) into five 40 mL pre-combusted amber glass vials. Samples were acidified with 0.1 mL of H3PO4 (85%) to remove inorganic C and stored in the dark at 4 °C until analysis. Dissolved organic carbon concentrations were measured using a total organic C analyzer (TOC-L; Shimadzu) (Ogawa et al., 1999). The instrument was calibrated with a standard addition curve of Potassium Hydrogen Phthalate (0; 16; 42; 67; 104; 167; 208 µmol C L−1). Consensus Reference Materials (CRM) provided by DA Hansell and W Chen of the University of Miami (Batch 22; 2022; DOC: 41–42 µmol L−1) were used as internal controls. DOC concentrations of the CRM gave average values (±SD) of 43.8 ± 1.2 µmol L−1.

Inorganic nutrients. Nitrate, nitrite, phosphate, and silicate concentrations were measured using a segmented flow analyzer (Model AA3 HR, SEAL Analytical IC) (Grasshoff et al., 2009; Murphy & Riley, 1962), whereas ammonium concentrations were analyzed fluorometrically (Turner Designs, Trilogy) following the procedure described in Molins-Legua et al. (2006).

References

Grasshoff, K., Kremling, K., & Ehrhardt, M. (2009). Methods of Seawater Analysis. John Wiley & Sons.

Molins-Legua, C., Meseguer-Lloret, S., Moliner-Martinez, Y., & Campíns-Falcó, P. (2006). A guide for selecting the most appropriate method for ammonium determination in water analysis. Trends in Analytical Chemistry, 3(25), 282–290. https://doi.org/10.1016/j.trac.2005.12.002

Murphy, J., & Riley, J. P. (1962). A modified single solution method for the determination of phosphate in natural waters. Analytica Chimica Acta, 27, 31–36. https://doi.org/10.1016/S0003-2670(00)88444-5

Ogawa, H., Fukuda, R., & Koike, I. (1999). Vertical distributions of dissolved organic carbon and nitrogen in the Southern Ocean. Deep Sea Research Part I: Oceanographic Research Papers, 46(10), 1809–1826. https://doi.org/10.1016/S0967-0637(99)00027-8

ESM Figure S2: *In situ* water temperature during the experimental period

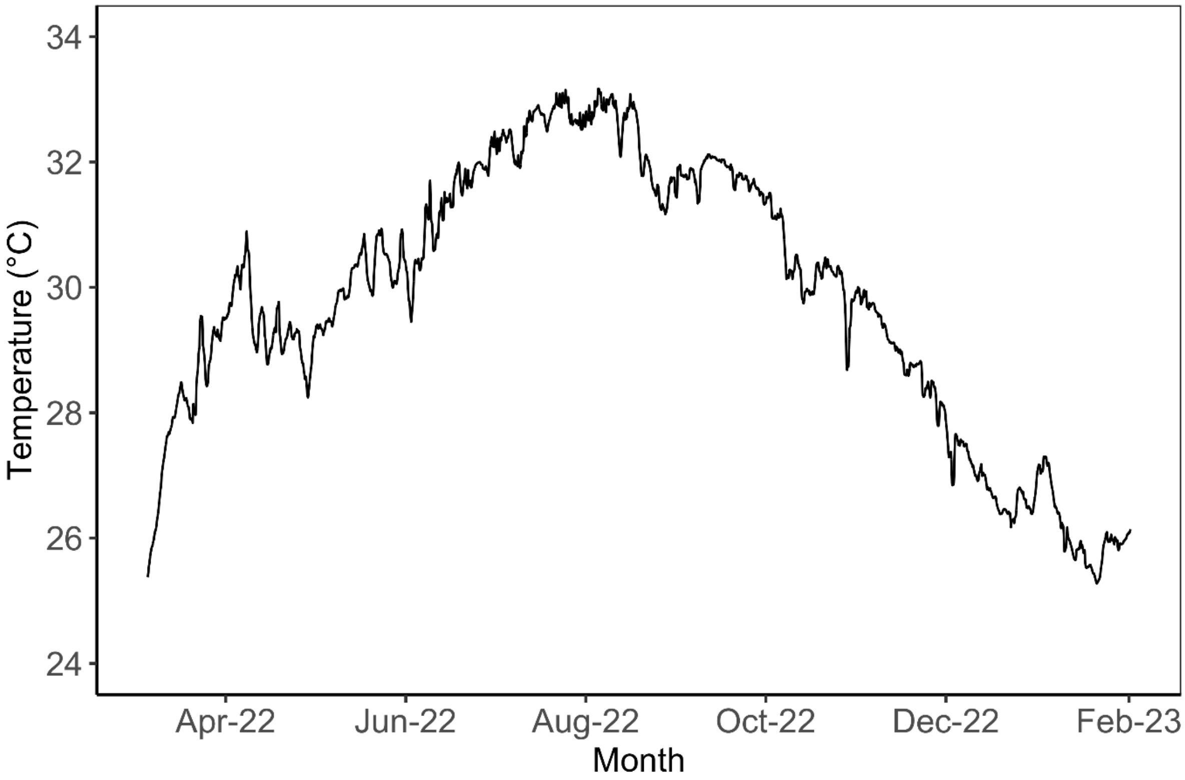

In situ temperature obtained by a temperature logger (Onset Hobo pendant) deployed at 1 m water depth at Al Fahal Reef (22.30518N, 38.96468E) in the central Red Sea. Temperature was measured in 10 min time intervals and averaged per day during the entire experimental period (April 2022 – February 2023).

ESM Text S3: Separation of host tissue from Symbiodiniaceae cells for stable isotope analyses

Coral tissue slurry was defrosted on ice for 30 min and pulse-vortexed for 5-10 seconds to ensure a homogenized mixture, then aliquoted into three 3 mL Eppendorf tubes (triplicates, tube A). Tubes were centrifuged for 2.5 min at 500 x g (the pellet containing Symbiodiniaceae cells) and the supernatant containing host tissue was transferred into a new tube (tube B) which was stored at -20 °C for processing of host tissue. When removing the host-containing supernatant from the Eppendorf (tube A), the supernatant was pipetted carefully from the upper surface to avoid disturbing the algal pellet. A small residual layer of host supernatant was intentionally left above the pellet to minimize the risk of resuspending or dislodging the algal cells. To isolate and clean the Symbiodiniaceae fraction, the pellet in tube A was washed two times in 3 mL MilliQ (i.e., vortex for 5-10 seconds, centrifuge at 400 x g for 2 min, discard supernatant). The final pellet was resuspended in 3 mL MilliQ (tube A), the presence of Symbiodiniaceae was confirmed using light microscopy, and the suspension was stored at -20 °C until further processing. Tubes A and B containing Symbiodiniaceae and host tissue fractions, respectively, were defrosted, vortexed for 5-10 seconds, and filtered over glass fiber filters (GF/F, 0.7 µm pore size) using a vacuum pump. To dissolve skeleton contaminants, 2 mL of 1M HCl were pipetted onto each filter, followed by 2 mL of MilliQ water to remove the acid. Filtration equipment, tweezers and work bench were cleaned with 10% bleach solution and rinsed with MilliQ between samples. Filters were placed into sterile falcon tubes (open lid) and dried at 60 °C for 48 h. Dried tissue was scraped off each filter with a sterile blade and tweezers and transferred into tin cups which were placed into a 96-well plate for analyses. Samples in April with C or N content < 0.015 mg were considered to have too low mass and the C:N ratio, as well as δ13C or δ15N, respectively, were excluded from subsequent analyses.

ESM Text S4: Adapted DNA extraction using DNeasy Blood and Tissue Kit and PCR

Coral fragments (1 cm) from the -80°C freezer (see "sample collection") were cut into smaller pieces using sterile pliers and placed into eppendorf tubes. A volume of 180 μL of ATL buffer was added. Next, 20 μL of proteinnase K was added, and samples were vortexed for 5-10 seconds, and incubated overnight in a thermomixer (ThermoFisher, 56°C at 650 rpm). The next morning, DNA purification and extraction steps of the protocol were followed. Extracted DNA was stored at -80 °C. Each 25 μL PCR reaction contained 12.5 μL 2X KAPA HiFi HotStart ReadyMix (final 1X), 1.0 μL forward primer 341F (10 μM; 0.3 μM final), 1.0 μL reverse primer 805R (10 μM; 0.3 μM final), 2.0 μL template DNA, and 8.5 μL water; thermal cycling conditions was set as 95 °C for 3 min (initial denaturation); followed by 25 cycles of 98 °C for 20 s (denaturation), 54 °C for 60 s (annealing), 72 °C for 90 s (extension); with a final 72 °C for 5 min (final extension).

ESM Text S5: Processing of demultiplexed raw sequence reads using the Dada2 package

Raw sequence reads were truncated manually to remove primers and quality filtered using the trimandFilter() command with the following parameters: trimLeft = c(17,21), truncLen = c(270,225), maxN=0, maxEE=c(2,2), truncQ=2. The error learning step of the dada2 pipeline was conducted with nbases = 1 x 108. Errors were removed, and the sequence composition was determined using the dada2() function. Error-corrected paired-end sequences were merged with a minimum of a 12 base pair overlap region and zero mismatches. Merged sequences aligning with the expected bimodal length distribution were retained by discarding all merged sequences less than 400 bp and greater than 430 bp. Chimeras were removed using the removeBimeraDenovo() function, with method = “consensus”. The sequence table was then used for taxonomic assignments against the Silva V138 (Quast et al., 2013) database.

References

Quast, C., Pruesse, E., Yilmaz, P., Gerken, J., Schweer, T., Yarza, P., Peplies, J., & Glöckner, F. O. (2013). The SILVA ribosomal RNA gene database project: Improved data processing and web-based tools. Nucleic Acids Research, 41(D1), D590–D596. https://doi.org/10.1093/nar/gks1219

ESM Figure S6: Refractory curve of bacterial taxa associated with *M. dichotoma* and *S. pistillata*

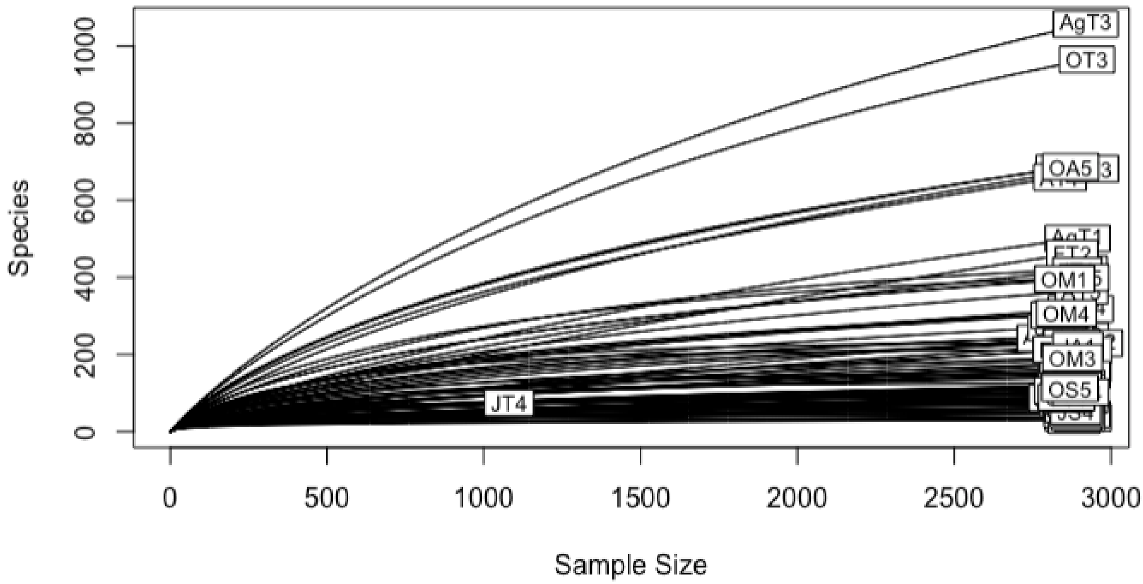

Rarefaction curve of all 16S rRNA amplicon sequencing samples produced by the rarecurve command in phyloseq.

ESM Text S7: Standard statistical analyses

Stable isotope- and molar C:N ratio data were analyzed per species with linear mixed-effects models (*lmer* package; Season, Tissue type, Season x Tissue type, random intercept ColonyID) to account for correlation of host-symbiont pairs and unbalanced pairing . Homogeneity of variance and normal distribution were confirmed visually (*performance* package). Homogeneity of variance was not given for δ^{15}N values of *S. pistillata* (i.e., significantly lower variance in Symbiodiniaceae- relative to host tissue in spring and summer), but model results were still reported for consistency, as the same outcomes were observed when fitting a model with weighted variances (i.e., heteroscedastic model with type-specific residual variance; *nlme varIdent*) . Pairwise comparisons for significant main effects were obtained with emmeans (false discovery rate-adjusted). Outliers (*rstatix* package) were removed before analyses and April C:N measurements were excluded due to a batch artifact.

We tested for correlations between host and Symbiodiniaceae δ^{13}C, δ^{15}N and C:N values, as well as between stable isotope- and elemental ratios with environmental variables using Spearman correlation tests (fdr-corrected *P* values; $R^2 > 0.5$) for each species.

The effects of species and season on bacterial community compositions (genus-level) and predicted metabolic functions (level of predicted functional groups) were assessed through two-way permutational multivariate ANOVA (PERMANOVA, adonis2 function, *vegan* package (Dixon, 2003)) on square root-transformed data. When significant, pairwise comparisons of seasons (three one-way PERMANOVAs) were performed using *pairwiseAdonis* package (i.e., summer did not significantly differ from winter and/or summer and was not considered for compositional nor pathway enrichment analyses). Similar multivariate distribution among groups was confirmed with betadisper. PERMANOVA P values were corrected for multiple comparisons using Bonferroni adjustment.

Multidimensional distribution was visualized using canonical analysis of principle coordinates (capscale function of the *vegan* package) based on Bray-Curtis dissimilarity on square root-transformed data. Co-linear environmental variables (i.e., testing DIN, DOC, Silica, Phosphate, PAR, chlorophyll *a* and SST) were excluded. For significantly different seasons, differential expression analysis (*DESeq2* package) was used to test which bacterial taxa and predicted functional groups differed significantly (Love et al., 2014)).

References

Dixon, P. (2003). *VEGAN*, a package of R functions for community ecology. *Journal of Vegetation Science*, *14*(6), 927–930. https://doi.org/10.1111/j.1654-1103.2003.tb02228.x

Love, M. I., Huber, W., & Anders, S. (2014). Moderated estimation of fold change and dispersion for RNA-seq data with DESeq2. Genome Biology, 15(12), 550. https://doi.org/10.1186/s13059-014-0550-8

ESM Table S8: PERMANOVA results of species and season effects for bacterial community compositions and metabolic functions

Bold values indicate significant effect (Bonferroni-adjusted $P < 0.05$). Data was square root transformed. Pairwise comparisons of seasons (Spring: April and June 2023; Summer: August and October 2023; Winter December 2024 and February 2024) for *M. dichotoma* were done with the "*pairwiseAdonis*" package. Similar multivariate distribution among groups was tested with *betadisper*.

Factor	df	F	R^2	P	P adj. (bonf.)
**Composition (genus)**					
Species	1	52.7	0.46	0.001	**0.008**
Season	2	3.5	0.06	0.002	**0.016**
Species * Season	2	3.5	0.06	0.005	**0.040**
M. dichotoma: Season	2	2.5	0.16	0.006	**0.048**
Spring vs. Winter	1	3.6	0.18	0.001	**0.008**
Summer vs. Spring	1	2.5	0.12	0.022	0.176
Summer vs. Winter	1	1.6	0.09	0.124	0.992
S. pistillata: Season	2	1.1	0.09	0.305	1.000
**Predicted function**					
Species	1	148.8	0.72	0.001	**0.003**
Season	2	2.2	0.02	0.09	0.27
Species * Season	2	2.3	0.02	0.08	0.24

ESM Figure S9: Bacterial taxa associated with *M. dichotoma* and *S. pistillata*

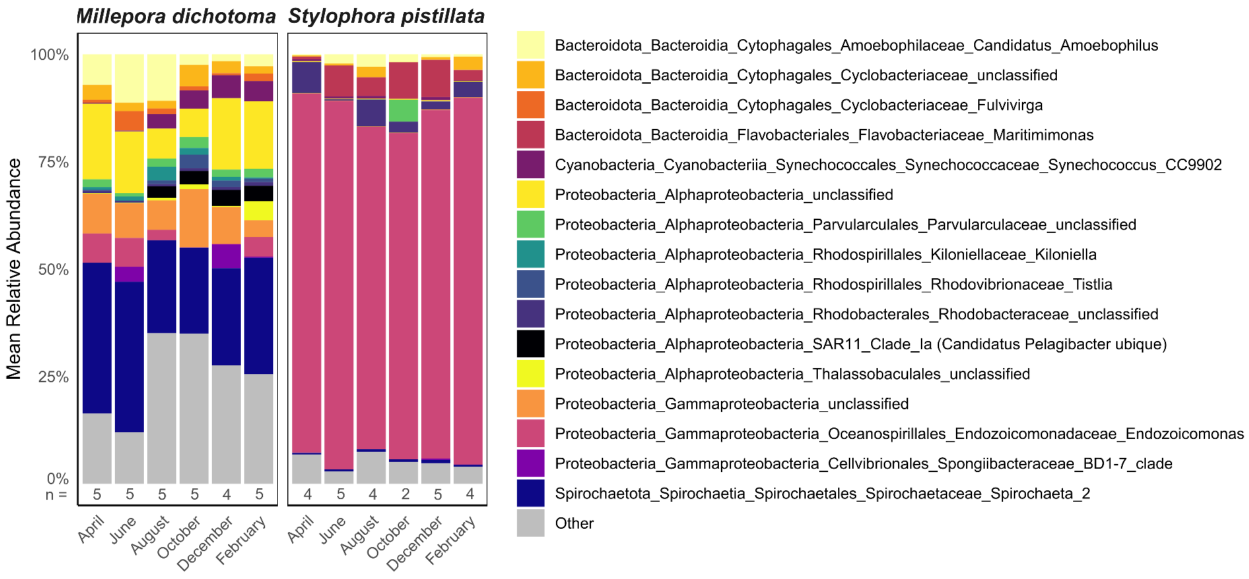

Bacterial taxa at the genus level associated with the corals *M. dichotoma* and *S. pistillata* in spring (April and June), summer (August and October) and winter (December and February). Only genera with more than 3% mean relative abundance for one species x month combination. Two outliers were excluded for in *S. pistillata* bacterial communities (one in April “AS2” and one in October “OS4”) for better visual representation.

ESM Figure S10: Representative pictures of the studied species taken at the sampling site.

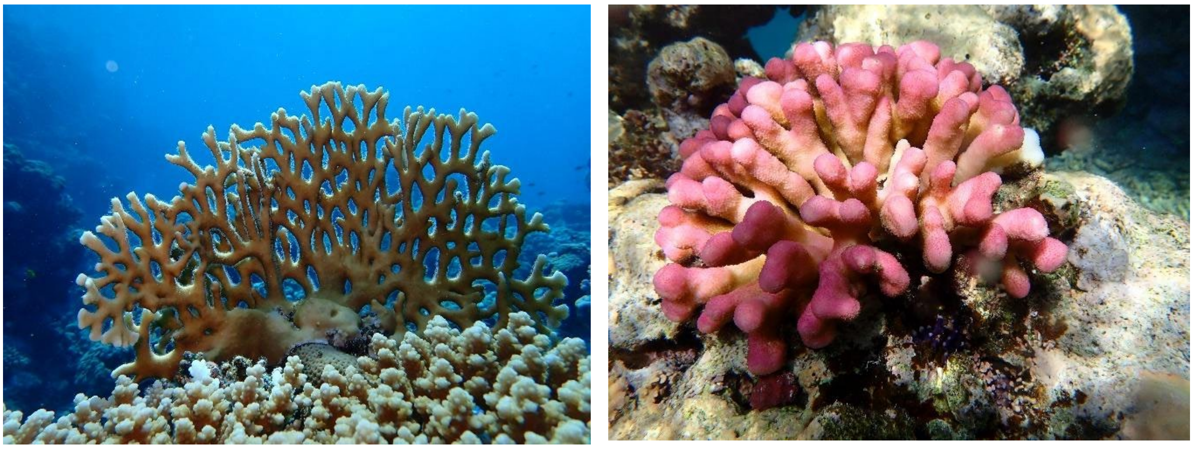

Left: *Millepora dichotoma*, right: *Stylophora pistillata*. One exposed fragment of ~ 5 cm length was used for C/N analyses and one fragment of ~1 cm length was used for bacterial community analyses. Photographs taken by Vivian Bonacker.
